# Prognostic implications of post-percutaneous coronary intervention neutrophil-to-lymphocyte ratio on infarct size and clinical outcomes in patients with acute myocardial infarction

**DOI:** 10.1038/s41598-019-46117-8

**Published:** 2019-07-04

**Authors:** David Hong, Ki Hong Choi, Young Bin Song, Joo Myung Lee, Taek Kyu Park, Jeong Hoon Yang, Joo-Yong Hahn, Jin-Ho Choi, Seung-Hyuk Choi, Sung Mok Kim, Yeonhyeon Choe, Eun Kyoung Kim, Sung A. Chang, Sang-Chol Lee, Jae K. Oh, Hyeon-Cheol Gwon

**Affiliations:** 1 Division of Cardiology, Department of Internal Medicine, Heart Vascular Stroke Institute, Samsung Medical Center, Sungkyunkwan University School of Medicine, Seoul, Republic of Korea; 2 Department of Critical Care Medicine, Samsung Medical Center, Sungkyunkwan University School of Medicine, Seoul, Republic of Korea; 3 Department of Emergency Medicine, Samsung Medical Center, Sungkyunkwan University School of Medicine, Seoul, Republic of Korea; 4 Department of Radiology, Heart Vascular Stroke Institute, Samsung Medical Center, Sungkyunkwan University School of Medicine, Seoul, Republic of Korea; 5 Cardiovascular Imaging Center, Heart Vascular Stroke Institute, Samsung Medical Center, Sungkyunkwan University School of Medicine, Seoul, Republic of Korea; 6 Division of Cardiovascular Diseases, Mayo Clinic College of Medicine, Rochester, MN USA

**Keywords:** Risk factors, Interventional cardiology

## Abstract

This study evaluated the prognostic implications of post-percutaneous coronary intervention (PCI) neutrophil-to-lymphocyte ratio (NLR) in patients with acute myocardial infarction (AMI). A total of 309 patients with AMI who underwent cardiac magnetic resonance imaging (CMR) and a complete blood cell count within 24 hours before and after PCI were enrolled. Primary outcome was infarct size. Patients were assigned to high (n = 118) or low (n = 191) NLR groups according to the best cut-off value of 3.88. Infarct size (% of total left ventricular mass) was significantly higher in the high NLR group than in the low NLR group (24.1 ± 11.0 vs. 16.7 ± 9.1, p < 0.001). Post-PCI NLR ≥ 3.88 was associated with risk of a large-sized infarction (≥20%) (OR 2.91, 95% CI 1.73–4.88, p < 0.001). The risk of MACE was also significantly higher in the high NLR group than in the low NLR group (15.8% vs. 7.4%, HR 2.60, 95% CI 1.21–5.60, p = 0.015). Among patients with AMI who underwent PCI, high post-PCI NLR value was associated with higher risk of large-sized infarction as measured by CMR, as well as adverse clinical outcomes. Our findings suggest that post-PCI NLR is a useful tool for risk assessment in patients with AMI who undergo PCI.

## Introduction

Inflammation plays a role in the initiation and progression of the atherosclerotic process, which is a leading cause of coronary artery disease^1,2^. Inflammation also occurs as a result of acute myocardial infarction (AMI) and plays a role in the repair and remodeling of infarcted heart tissue^3^. Based on this concept, several studies have investigated the associations between various inflammatory markers and clinical outcomes in patients with coronary artery disease^4–8^. Neutrophil-to-lymphocyte ratio (NLR) has emerged as an independent predictor of cardiovascular outcomes in patients with ischemic heart disease, including AMI^9–15^. Especially, post-percutaneous coronary intervention (PCI) NLR was found to be a better indicator of severity of myocardial damage than pre-PCI NLR^12–14^.

Cardiac magnetic resonance imaging (CMR) is the current gold-standard technique for evaluating infarct size and transmural extent of infarction and for performing microvascular assessment in the setting of AMI^16,17^. Furthermore, CMR allows quantification of the extent of area at risk and salvaged myocardium based on delayed hyperenhancement and T2-weighted images^16^. It is well known that infarct size has the strongest association with late systolic dysfunction and provides incremental prognostic information in addition to left ventricular ejection fraction^18,19^.

Though several studies have studied the relation between NLR and clinical outcomes, the associations between post-PCI NLR and structural markers of myocardial injury measured by CMR in patients with AMI are unknown. Therefore, we sought to evaluate the relationship between post-PCI NLR and infarct size, and between post-PCI NLR and clinical outcomes in patients with AMI.

## Methods

### Study population and data collection

**Figure 1:**
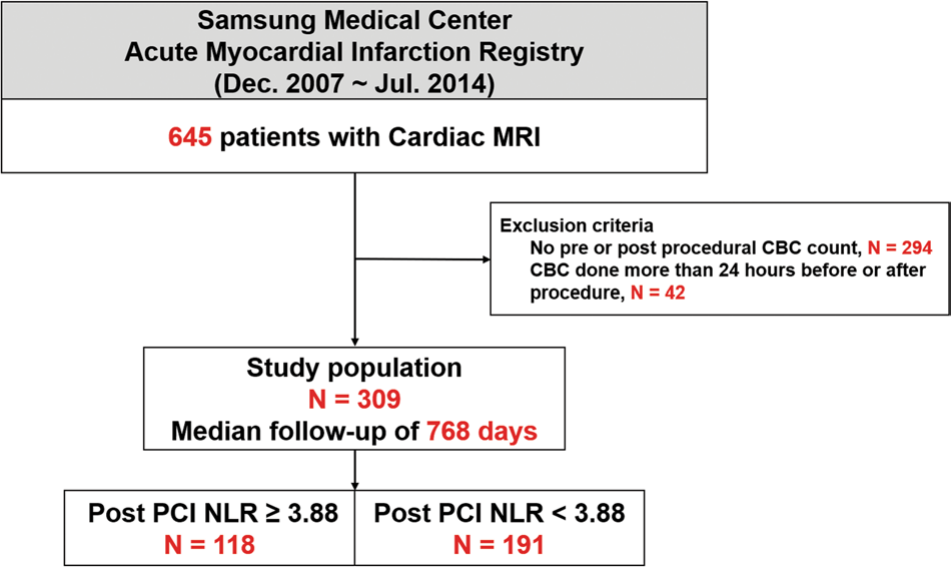
Study flow. Abbreviations: *CBC* complete blood cell count, *MRI* magnetic resonance imaging, *NLR* neutrophil-to-lymphocyte ratio, *PCI* percutaneous coronary intervention.

The study population was derived from the prospective institutional AMI registry of Samsung Medical Center between December 2007 and July 2014 (Fig. 1). AMI was defined as evidence of myocardial injury (defined as elevation of cardiac troponin values, with at least one value above the 99th percentile upper reference limit) with necrosis in a clinical setting, consistent with myocardial ischemia^20^. We excluded patients who did not undergo a complete blood cell count (CBC) within 24 hours before and after PCI. A total of 309 AMI patients who underwent CMR and had pre- and post-PCI CBC data was finally included in the current study (Fig. 1). The Institutional Review Board of Samsung Medical Center approved this study, and all patients provided written informed consent. All research was performed in accordance with relevant standard guidelines^20–23^.

Demographic features and cardiovascular risk factors were collected prospectively at the index procedure by patient interview. During hospitalization, coronary angiography, CMR, and echocardiography were conducted. After discharge, patients were followed-up by the attending physician based on individual clinical needs. Follow-up outcomes were obtained from medical records and telephone contact, if necessary. All data were collected by research coordinators of the dedicated registry. Clinical events that occurred within a 2-year follow-up period were analyzed.

### Treatment strategy

Patient management was performed according to relevant standard guidelines^20–23^. The choice of treatment strategy (type, diameter, and length of stents; use of intravascular ultrasound; glycoprotein IIb/IIIa inhibitor use; and thrombus aspiration) was at the discretion of the physicians. PCI was considered successful if the final residual stenosis was <30% with Thrombolysis In Myocardial Infarction grade 3 flow. All patients who were not taking aspirin or a P2Y12 inhibitor received a loading dose of aspirin or P2Y12 inhibitor, respectively. Unless there was an undisputed reason for discontinuing dual-antiplatelet therapy, all patients were recommended to take aspirin indefinitely plus a P2Y12 inhibitor for at least 1 year after the index procedure. Medications were prescribed according to standard guidelines.

### Cardiac magnetic resonance imaging

CMR was performed using a 1.5-T scanner (Achieva, Philips Medical Systems, Best, Netherlands) and analyzed using validated software (ARGUS, Siemens Medical System, Erlangen, Germany). Two experienced radiologists who were blinded to the clinical information measured CMR parameters using the software mentioned above (Supplementary Fig. 1). Infarct size and microvascular obstruction (MVO) were quantified using late gadolinium enhancement images^16^. Infarct size was calculated as summation of the area with delayed hyperenhancement within each segment of the short-axis images. This value was multiplied by slice thickness to cover the entire left ventricle. Endocardial and epicardial borders were then summed to calculate left ventricle myocardial volume using the same method. Infarct size was expressed as a percentage of the affected left ventricular myocardial volume. MVO, which was the extent of hypoenhancement within bright regions of late gadolinium enhancement, was calculated in the same manner^16^. Area at risk (AAR) was quantified on T2-weighted images using a similar algorithm as above^16^. From these parameters, myocardial salvage index (MSI) was derived as follows: MSI = (AAR-infarct size)/AAR^16^.

### Definitions and outcomes

The primary outcome was infarct size as measured by CMR after PCI. The main secondary outcome was 2-year major adverse cardiac events (MACE, a composite of all cause death, any myocardial infarction [MI], and any repeat revascularization). Other secondary outcomes were MVO, AAR, MSI as assessed by CMR, and each component of MACE. All clinical outcomes were defined according to the Academic Research Consortium^24^. All deaths were considered cardiac unless a definite non-cardiac cause could be established. Recurrent MI was defined as elevated cardiac enzymes greater than the upper limit of the normal value with ischemic symptoms or electrocardiography findings indicative of ischemia that were not related to the index procedure. Repeat revascularization was considered clinically indicated if there were symptoms or functional evidence of ischemia and/or lesion severity >50% of the diameter of stenosis by coronary angiography.

### Statistical analysis

Simple linear regression analysis was performed to determine the association between post-PCI NLR and infarct size. Receiver-operating-characteristic (ROC) curve analysis was performed to determine the best cut-off value of post-PCI NLR to predict a large-sized infarction as measured by CMR. A large-sized infarction was defined as an infarction involving ≥20% of the total left ventricular mass, which was a median value in a previous study^25^. The best cut-off value was used to divide the enrolled patients into the two groups.

Continuous variables were compared using Student’s t-test, and categorical variables were tested using the Chi-square test. Multivariable logistic regression analysis was performed to identify independent predictors of large myocardial infarct (≥20% of total left ventricular mass). We included in the multivariable models those covariates that were significant in univariate analysis or those that were clinically relevant.

Cumulative event rates of clinical outcomes were calculated Kaplan-Meier estimates, and significance levels were assessed with log-rank tests. Hazard ratios (HR) and 95% confidence intervals (CI) were calculated by Cox proportional hazards models to compare clinical outcomes that occurred after the index procedure, between the two groups. The time-to-event was measured as days in Cox proportional hazards models. Multiple sensitivity analyses, namely multivariable adjusted Cox proportional hazard regression and inverse-probability-weighted (IPW) analyses, were performed to adjust baseline differences in both groups. Standardized mean differences after IPW adjustment were within ±10% across all matched covariates, suggesting achievement of balance between the two groups (Supplementary Table 1).

All probability values were two-sided, and p-values < 0.05 were considered statistically significant. Statistical analyses were performed using SPSS (version 24.0, SPSS Inc., Chicago, IL, USA) and R Statistical Software (version 3.4.3, R Foundation for Statistical Computing, Vienna, Austria).

## Results

### Relation between post-PCI NLR and infarct size

**Figure 2:**
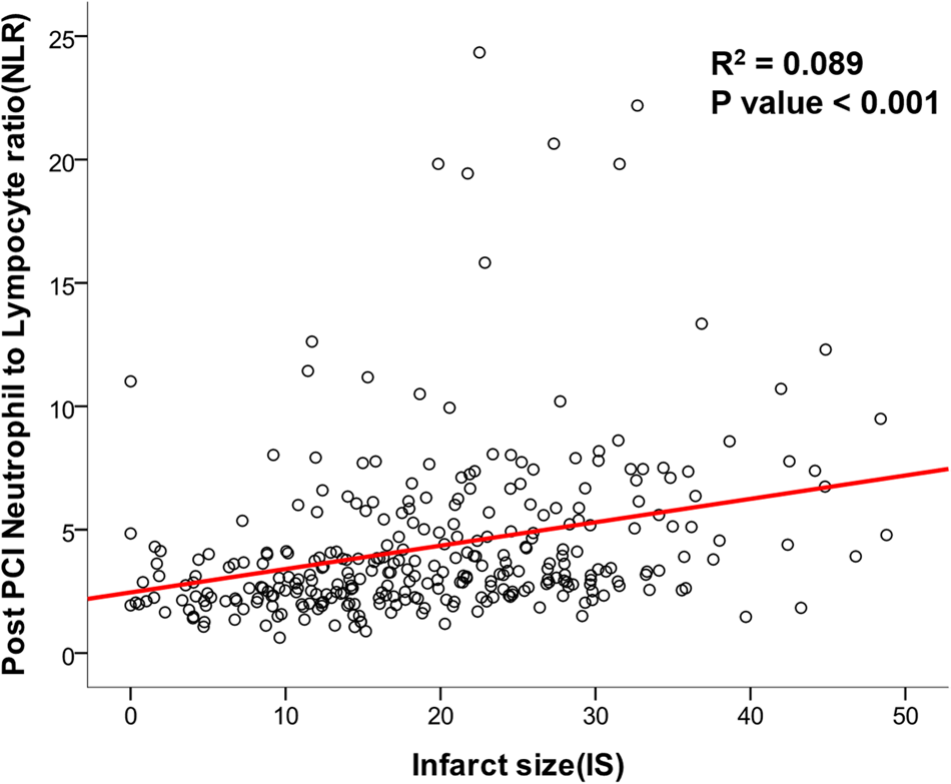
Relation between neutrophil-to-lymphocyte ratio (NLR) and infarct size (IS). Scatter plot is presented to show linear relation between post-PCI NLR and IS. Abbreviations: *IS* infarct size, *NLR* neutrophil-to-lymphocyte ratio, *PCI* percutaneous coronary intervention.

CMR was performed a median of 3.4 days (interquartile range 2.7–4.6 days) after the index procedure. To find which leukocytes and differential counts accurately distinguished large-sized (≥20% of the total left ventricular mass) infarctions, ROC curve analysis was performed. Total white blood cell counts and NLR before and after PCI were compared. Among these parameters, post-PCI NLR had the highest C-index (0.693, 95% CI 0.634–0.751) to discriminate a large-sized infarction, and the best cut-off value of post-PCI NLR was 3.88 (Supplementary Fig. 2). Linear regression analysis showed a significant association between post-PCI NLR and infarct size (R^2^: 0.089, p < 0.001) (Fig. 2).

### Baseline clinical and angiographic characteristics

According to the post PCI NLR value of 3.88, the study population was stratified into two groups: a high NLR group (post-PCI NLR ≥ 3.88, 118 patients) and a low NLR group (post-PCI NLR < 3.88, 191 patients). Baseline clinical and angiographic characteristics are shown in Tables 1 and 2. Patients in the high NLR group were older and had more frequent previous history of myocardial infarction and PCI than patients in the low NLR group. The high NLR group was more frequently manifested as ST-segment elevation myocardial infarction (STEMI) than those in the low NLR group. Other baseline characteristics were similar between the two groups.

**Table 1:** Baseline characteristics of patients with acute myocardial infarction according to an NLR cut-off value of 3.88.

Variables	Total (N = 309)	NLR ≥ 3.88 (N = 118)	NLR < 3.88 (N = 191)	P value
**Demographics**				
Age (years)	60.2 ± 12.3	62.1 ± 13.6	59.1 ± 11.3	0.045
Male	252 (81.6)	95 (80.5)	157 (82.2)	0.710
Body mass index, kg/m^2^	24.6 ± 3.4	24.0 ± 3.7	24.9 ± 3.1	0.024
**Cardiovascular risk factors**				
Hypertension	130 (42.1)	53 (44.9)	77 (40.3)	0.426
Diabetes mellitus	68 (22.0)	32 (27.1)	36 (18.8)	0.088
Dyslipidemia	51 (16.5)	16 (13.6)	35 (18.3)	0.273
History of myocardial infarction	15 (4.9)	10 (8.5)	5 (2.6)	0.020
History of percutaneous coronary intervention	22 (7.1)	14 (11.9)	8 (4.2)	0.011
History of cerebrovascular accident	14 (4.5)	6 (5.1)	8 (4.2)	0.713
**Laboratory findings**				
NT-proBNP, pg/mL	209.6 (55.9–807.5)	204.1 (69.1–1012.3)	212.6 (50.6–696.7)	0.246
White blood cell, ×10^3^/μL	9.9 ± 3.1	11.4 ± 3.4	9.0 ± 2.6	<0.001
Hemoglobin, g/dL	13.5 ± 1.9	13.2 ± 2.0	13.7 ± 1.8	0.013
Platelet, 10^3^/μL	206.8 ± 45.4	209.5 ± 48.6	205.1 ± 43.4	0.403
**Clinical presentation**				
NSTEMI	82 (26.5)	16 (13.6)	66 (34.6)	<0.001
Door-to-balloon time, min	681.5 (192.5–1287.5)	478.5 (155.5–1246.0)	700.0 (192.5–1308.3)	0.460
Symptom-to-balloon time, min	1296.0 (624.0–1892.8)	1265.5 (546.0–2468.5)	1338.5 (688.5–1892.8)	0.923
STEMI	227 (73.5)	102 (86.4)	125 (65.4)	<0.001
Door-to-balloon time, min	70.0 (53.0–82.0)	70.0 (51.0–81.5)	70.0 (53.5–82.0)	0.793
Symptom-to-balloon time, min	198.0 (116.0–410.0)	202.0 (101.5–412.5)	198.0 (123.0–423.0)	0.576
**Post-percutaneous coronary intervention medication**				
Aspirin	309 (100.0)	118 (100.0)	191 (100.0)	>0.99
P2Y12 inhibitor	303 (98.1)	116 (98.3)	187 (97.9)	>0.99
Beta-blocker	280 (90.6)	106 (89.8)	174 (91.1)	0.710
ACE inhibitor or ARB	246 (79.6)	89 (75.4)	157 (82.2)	0.151
Statin	296 (95.8)	111 (94.1)	185 (96.9)	0.255

Data are presented as n (%), mean ± SD, or median (interquartile range).

Abbreviations: *ACE* angiotensin converting enzyme, *ARB* angiotensin receptor blocker, *NLR* neutrophil-to-lymphocyte ratio, *NT-proBNP* N-terminal pro-brain natriuretic peptide, *STEMI* ST-segment elevation myocardial infarction, NSTEMI non-ST-segment elevation myocardial infarction.

**Table 2:** Angiographic and procedural characteristics of patients with acute myocardial infarction according to an NLR cut-off value of 3.88.

Variables	Total (N = 309)	NLR ≥ 3.88 (N = 118)	NLR < 3.88 (N = 191)	P value
Infarct-related artery				0.286
LAD	145 (46.9)	63 (53.4)	82 (42.9)	
LCX	48 (15.5)	16 (13.6)	32 (16.8)	
RCA	115 (37.2)	39 (33.1)	76 (39.8)	
LM	1 (0.3)	0 (0.0)	1 (0.5)	
Number of diseased vessels				0.253
1	163 (52.8)	56 (47.5)	107 (56.0)	
2	101 (32.7)	45 (38.1)	56 (29.3)	
3	45 (14.6)	17 (14.4)	28 (14.7)	
Multi-vessel disease	146 (47.2)	62 (52.5)	84 (44.0)	0.143
Pre-PCI TIMI flow ≤ 1	232 (75.1)	96 (81.4)	136 (71.2)	0.062
Post-PCI TIMI flow 3	288 (93.2)	108 (91.5)	180 (94.2)	0.357
Thrombus aspiration	166 (53.7)	69 (58.5)	97 (50.8)	0.188
Number of implanted stents	1.2 ± 0.7	1.1 ± 0.7	1.2 ± 0.6	0.833
Stent diameter, mm	3.2 ± 0.5	3.1 ± 0.5	3.2 ± 0.6	0.175
Stent diameter < 3 mm	122 (39.5)	55 (46.6)	67 (35.1)	0.058
Stent length, mm	31.6 ± 17.1	30.9 ± 16.9	32.0 ± 17.2	0.619

Data are presented as n (%), mean ± SD, or median (interquartile range).

Abbreviations: *LAD* left anterior descending artery, *LCX* left circumflex artery, *RCA* right coronary artery, *LM* left main coronary artery, *NLR* neutrophil-to-lymphocyte ratio, *PCI* percutaneous coronary intervention, *TIMI* thrombolysis in myocardial infarction.

### Outcomes

Laboratory, echocardiography, and CMR findings according to the NLR cut-off value of 3.88 are presented in Table 3. The high NLR group had a significantly larger infarct size than the low NLR group (24.1 ± 11.0 vs. 16.7 ± 9.1, p < 0.001). Size of AAR (40.7 ± 16.3 vs. 29.5 ± 15.1, p < 0.001) and MVO (2.9 [0–9.3] vs. 0 [0–3.7], p < 0.001), which are other important indicators of severe infarction, were also significantly larger in the high NLR group. MSI tended to be lower in the high NLR group than the low NLR group (40.3 ± 16.9 vs. 42.7 ± 17.5, p = 0.237), but this difference was not statistically significant. Post-PCI NLR ≥ 3.88 was the strongest predictor of large-sized infarction (odds ratio: 2.91, 95% CI: 1.73–4.88, p < 0.001) by multivariable logistic regression analysis (Table 4).

**Table 3:** Infarct size assessment using laboratory, echocardiography, and cardiac MRI data according to an NLR cut-off value of 3.88.

Variables	Total (N = 309)	NLR ≥ 3.88 (N = 118)	NLR < 3.88 (N = 191)	P value
**Cardiac laboratory profiles after percutaneous coronary intervention**				
Peak troponin I, ng/mL	45.3 (11.9–116.2)	75.3 (22.3–185.5)	35.8 (8.0–80.3)	<0.001
Peak CK-MB, ng/mL	140.3 (47.8–248.9)	193.2 (80.0–314.0)	100.8 (39.3–210.9)	<0.001
**Echocardiography**				
EF	52.2 ± 10.9	49.1 ± 11.5	54.1 ± 10.0	<0.001
Wall motion score index (WMSI)	1.4 (1.2–1.7)	1.5 (1.2–1.8)	1.3 (1.1–1.6)	<0.001
**Cardiac MRI**				
Infarct size (% of LV)	19.5 ± 10.5	24.1 ± 11.0	16.7 ± 9.1	<0.001
Area at risk (% of LV)	33.8 ± 16.5	40.7 ± 16.3	29.5 ± 15.1	<0.001
Myocardial salvage index	41.8 ± 17.3	40.3 ± 16.9	42.7 ± 17.5	0.237
Microvascular obstruction (per Vol)	0.9 (0–5.4)	2.9 (0–9.3)	0 (0–3.7)	<0.001
LV EDV	148.3 ± 37.2	149.7 ± 41.5	147.5 ± 34.3	0.612
LV ESV	73.3 ± 32.9	79.8 ± 40.3	69.3 ± 26.7	0.013
LV mass (g)	108.7 ± 27.4	107.9 ± 29.5	109.2 ± 26.1	0.700
LV EF	52.1 ± 10.8	48.8 ± 11.7	54.2 ± 9.7	<0.001
LV SV	74.8 ± 16.9	69.9 ± 16.0	77.9 ± 16.7	<0.001
LV CO	5.1 ± 1.1	5.0 ± 1.1	5.2 ± 1.1	0.172

Data are presented as mean ± SD or median (interquartile range).

Abbreviations: *CK-MB* creatine kinase-myocardial band, *CO* cardiac output, *EDV* end diastolic volume, *EF* ejection fraction, *ESV* end systolic volume, *LV* left ventricle, *NLR* neutrophil-to-lymphocyte ratio, *SV* stroke volume.

**Table 4:** Independent predictors of large myocardial infarction (≥20%) in patients with acute myocardial infarction.

	OR (95% CI)*	P value
ST-segment elevation myocardial infarction	1.81 (1.02–3.24)	0.042
Post PCI NLR ≥ 3.88	2.91 (1.73–4.88)	<0.001
Male	1.98 (1.01–3.87)	0.043
Body mass index ≥ 25 kg/m^2^	0.55 (0.33–0.92)	0.021

Adjusted variables were age, male, hypertension, diabetes mellitus, history of myocardial infarction, history of PCI, ST-segment elevation myocardial infarction, multi-vessel disease, anterior infarction, body mass index ≥ 25 kg/m^2^, and post-PCI NLR ≥ 3.88.

*C-index of the logistic regression model for large infarct size was 0.717 (95% CI 0.659–0.775).

Abbreviations: *CI* confidence interval, *OR* odds ratio, *NLR* neutrophil-to-lymphocyte ratio, *PCI* percutaneous coronary intervention, *TIMI* Thrombolysis In Myocardial Infarction.

**Figure 3:**
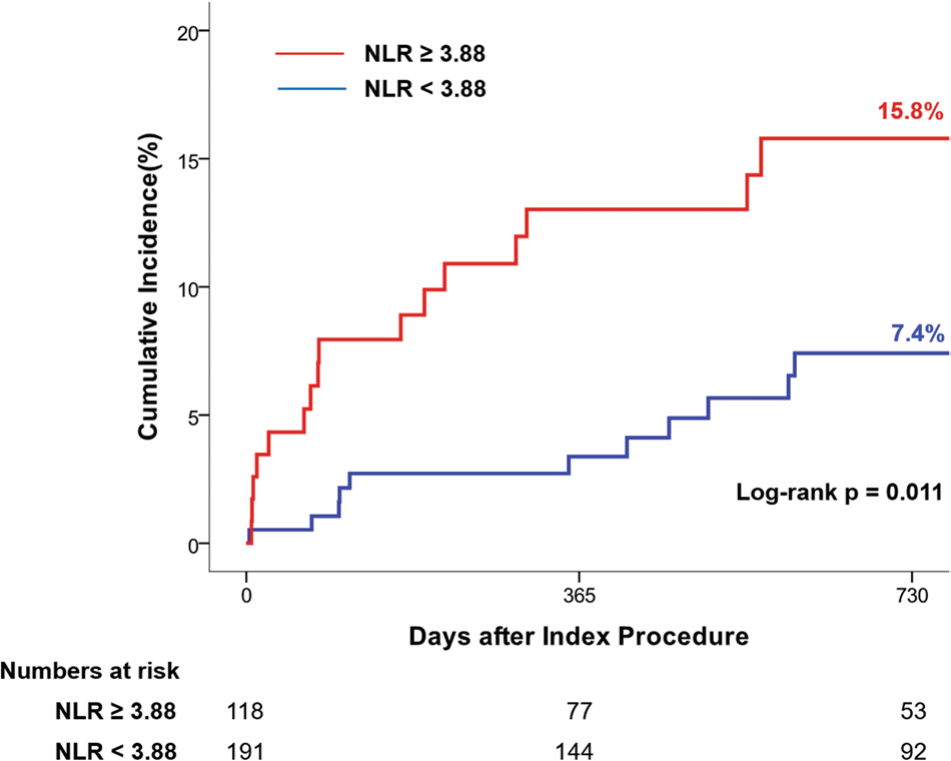
Cumulative incidence of major adverse cardiac events (MACE) at 2 years after the index procedure. Kaplan-Meier curves are presented to compare the cumulative incidence of MACE between the high NLR group and low NLR group. Abbreviations: *MACE* major adverse cardiac event, *NLR* neutrophil-to-lymphocyte ratio.

Median follow-up duration was 768 days. Cumulative incidence of MACE was significantly higher in the high NLR group than in the low NLR group (15.8% vs. 7.4%, HR: 2.60, 95% CI: 1.21–5.60, p = 0.015) (Table 5 and Fig. 3). After adjusting baselines by multivariable Cox regression and IPW adjustment, post-PCI NLR ≥ 3.88 was consistently associated with a higher risk of MACE (Table 5). The higher risk of MACE in AMI patients with high post-PCI NLR was mainly driven by the higher rates of hard endpoints, including all-cause death (4.6% vs. 0.6%, HR 8.47, 95% CI 0.99–72.51, p = 0.051) and MI (4.9% vs. 0.8%, HR 8.64, 95% CI 1.01–73.97, p = 0.049) (Table 5 and Supplementary Fig. 3).

**Table 5:** Two-year clinical outcomes in patients with acute myocardial infarction according to an NLR cut-off value of 3.88.

	NLR ≥ 3.88 (n = 118)	NLR < 3.88 (n = 191)	Unadjusted		Adjusted		IPW-adjusted	
			HR(95% CI)	p value	HR(95% CI)	p value	HR(95% CI)	p value
MACE	16 (15.8)	11 (7.4)	2.60 (1.21–5.60)	0.015	2.38 (1.01–5.17)	0.028	1.69 (1.00–2.86)	0.048
All-cause death	5 (4.6)	1 (0.6)	8.47 (0.99–72.51)	0.051	6.09 (0.70–52.78)	0.101	2.65 (0.96–7.26)	0.059
Myocardial infarction	5 (4.9)	1 (0.8)	8.64 (1.01–73.97)	0.049	6.70 (0.75–59.70)	0.088	4.31 (0.88–21.18)	0.072
Any revascularization	8 (8.8)	8 (5.5)	1.78 (0.67–4.74)	0.250	1.50 (0.53–4.25)	0.445	1.30 (0.65–2.57)	0.456

Adjusted variables were age, sex, diabetes mellitus, history of myocardial infarction, ST-segment elevation myocardial infarction, anterior infarction, TIMI flow grade 0 before PCI.

Data are presented as n (%).

Abbreviations: *CI* confidence interval, *HR* hazard ratio, *IPW* inverse-probability-weighted, *MACE* major cardiac adverse event, *NLR* neutrophil-to-lymphocyte ratio, *PCI* percutaneous coronary intervention, *TIMI* Thrombolysis In Myocardial Infarction.

### Subgroup analysis of STEMI patients

Because presentation with STEMI is an independent predictor of large-sized infarction, subgroup analysis was performed to determine whether post-PCI NLR plays a role in risk stratification for STEMI patients. STEMI patients with NLR ≥ 3.88 had significantly larger infarct size, AAR, and MVO, consistent with findings in the larger study population (Supplementary Fig. 4). In addition, the high post-PCI NLR group showed significantly higher risk of MACE than the low NLR group among patients who presented with STEMI (Supplementary Fig. 5).

## Discussion

In this present study, we evaluated the association between post-PCI NLR and infarct size as well as clinical outcomes in patients with AMI. Our key findings were as follows. First, among various pre- and post-leukocyte parameters and differential counts, post-PCI NLR showed the best ability to discriminate large-sized infarctions. Second, elevated post-PCI NLR was associated with larger infarct size and MVO and higher risk of MACE, and this association was robust after adjusting baselines using multivariable adjusted Cox regression and IPW adjustments. Third, subgroup analysis of STEMI patients revealed that infarct size was significantly larger and risk of MACE significantly higher in the post-PCI NLR ≥ 3.88 group than the post-PCI NLR < 3.88 group.

AMI remains one of the most important causes of morbidity and mortality worldwide despite recent advances in PCI techniques and devices^26^. Therefore, there have been numerous efforts to predict prognosis and improve outcomes in AMI patients^18,27,28^. Inflammation is not only an important cause of atherosclerosis, but also involved in the repair and adverse remodeling of infarcted heart tissue^3^. In this regard, interest in the role of inflammation in patients with AMI is increasing, and numerous studies have evaluated the associations between various inflammatory markers and clinical outcomes in patients with AMI^4–15^. A previous study showed that leukocyte differentials have greater predictive value of clinical outcome than total leukocyte count^29^. In that study, NLR was the most powerful predictor of death or myocardial infarction among various leukocyte parameters and differential counts^29^. In particular, Park *et al*. showed that post-PCI NLR was more strongly associated with mortality than baseline or pre-PCI NLR^12^. A possible explanation for this finding is that the time elapsed after infarction is too short for pre-PCI leukocyte profiles to fully reflect the severity of the ongoing myocardial damage. Similarly, considering that CMR was performed few days after PCI, temporal proximity may be a reason to explain that the post-PCI NLR may better reflect infarct size than the pre-PCI NLR. Furthermore, post-PCI leukocyte profiles have the advantage that they may also reflect peri-procedural myocardial damage^14^. In accordance with previous studies^12,14,29^, we showed that patients with AMI and a high post-PCI NLR had a higher risk of adverse cardiac events than those with a low post-PCI NLR. The reason for this association is that, in addition to their role in repair of the infarcted heart, leukocytes are also involved in infarct expansion^29,30^. After infarction, neutrophils release superoxide radicals, enzymes, and other metabolites that facilitate plaque disruption and lead to infarct expansion. Neutrophils aggregate with platelets and damage micro-vessels, which can also promote infarct expansion^29^. In addition, lymphocytes are trapped and sequestered within the myocardial microvasculature and release inflammatory mediators, contributing to further leukocyte infiltration and myocardial damage^31^. On this background, the results of the current study suggest that post-PCI NLR might be a predictor of clinical outcomes in AMI patients.

CMR is considered the gold standard for evaluating cardiac volumes and systolic function^16^. In addition, CMR has become the most accurate tool to visualize and quantify post-infarction parameters in patients with AMI^18^. Among post-infarction parameters, infarct size is strongly associated with mortality and hospitalization for heart failure^32^. Furthermore, infarct size can provide incremental prognostic information in addition to left ventricular ejection fraction, which is a well-established predictor of outcome in AMI^18^. Because left ventricular ejection fraction is influenced both by stunned viable myocardium and nonviable myocardium, CMR parameters can be more specific markers for determining the extent of irreversible myocardial damage^33^. Although several studies have investigated the association between white blood cell count and extent of infarct measured by CMR^4,34–36^, limited data are available regarding the association between post-PCI NLR and infarct size. Therefore, we sought to assess whether high post-PCI NLR value is associated with larger infarct size as assessed by CMR in patients with AMI. We found that high post-PCI NLR was consistently associated with larger infarct size and MVO as assessed by various tools, including cardiac enzymes, echocardiography, and CMR in patients with AMI who underwent PCI. Furthermore, there was a linear correlation between post-PCI NLR and infarct size as measured by CMR. Although the myocardial salvage index was numerically lower in the high post-PCI NLR group, this difference was not statistically significant. The results of our study suggest that post-PCI NLR, which is a simply measured marker of inflammation, can help predict infarct size as well as prognosis of patients with AMI after PCI.

In the current study, we thoroughly compared various pre and post PCI leukocytes profiles and identified that post-PCI NLR showed the best ability to discriminate large-sized infarctions assessed by CMR. To verify prognostic implication of post-PCI NLR, comprehensive assessment was conducted to find relation with post-PCI NLR and various markers of infarct size such as laboratory and echocardiographic findings as well as adverse clinical outcomes. Furthermore, the robustness of these results were supported by many sensitivity analyses such as multivariable regression and IPW adjustment. Thereby our results suggest that post-PCI NLR can be used for predicting prognosis as well as an infarct size predictor in future trials that aim to reduce infarct size. However, this study had several limitations. First, it was a retrospective study. Therefore, there may have been selection bias, and confounding variables may not have been accounted for. To overcome retrospective design of study, multivariable regression and IPW adjustment were performed to minimize the effect of confounders. Second, many patients were excluded from the study because they did not undergo pre- and post-PCI CBC with differential counts. This exclusion could have resulted in selection bias. Third, the number of patients who presented with STEMI was significantly different between the low and high post-PCI NLR groups. However, results from subgroup analysis of STEMI patients only were consistent with the results obtained for the entire study population. Fourth, the post-PCI NLR cut-off value was lower than that reported in previous studies^12,13^. Unlike previous studies that reported NLR cut-off values that best predicted clinical outcomes, we determined the cut-off value that best predicted large-sized infarction. Therefore, it is not surprising that the cut-off value was lower than reported in previous studies because not all patients who have a large-sized infarction have clinical events. Our cut-off value showed a significant association with poorer clinical outcomes and large-sized infarction.

## Conclusions

High post-PCI NLR value was associated with higher risk of large-sized infarction as measured by CMR, as well as adverse clinical events in AMI patients who underwent PCI. Our findings suggest that post-PCI NLR, which is an easily measurable and universally available marker, could be a useful tool for risk assessment and prognosis prediction in AMI patients undergoing PCI.

## Supplementary information

Supplementary Appendix

## Data Availability

The data that support the findings of this study are available, on reasonable request, from the corresponding author.
