# Supplementary material for: Prognostic implications of post-percutaneous coronary intervention neutrophil-to-lymphocyte ratio on infarct size and clinical outcomes in patients with acute myocardial infarction
Source: Sci Rep. 2019 Jul 4;9:9646. doi: 10.1038/s41598-019-46117-8 (PMC6609693; doi:10.1038/s41598-019-46117-8)
Supplement: Supplementary file 1 — Supplementary Appendix [file 41598_2019_46117_MOESM1_ESM.pdf]

Supplementary appendix

Prognostic implication of post-percutaneous coronary intervention neutrophil-to-lymphocyte ratio on infarct size and clinical outcomes in patients with acute myocardial infarction

David Hong, MD[1†], Ki Hong Choi, MD[1†], Young Bin Song, MD, PhD[1*], Joo Myung Lee, MD, MPH, PhD[1], Taek Kyu Park, MD, PhD[1], Jeong Hoon Yang, MD, PhD[1,2], Joo-Yong Hahn, MD, PhD[1], Jin-Ho Choi, MD, PhD[1,3], Seung-Hyuk Choi, MD, PhD[1], Sung Mok Kim, MD, PhD[4], Yeonhyeon Choe, MD, PhD[4], Eun Kyoung Kim, MD, PhD[5], Sung A Chang, MD, PhD[5], Sang-Chol Lee, MD, PhD[5], Jae K. Oh, MD, PhD[5,6], Hyeon-Cheol Gwon, MD, PhD[1]

Table of contents

(1) Supplementary Tables

(2) Supplementary Figures and Figure legends

(1) Supplementary Tables

Supplementary Table 1. Standardized differences of variables used in IPW adjustment to adjust baseline differences between the NLR≥3.88 and NLR<3.88 group

	Standardized differences, %	
	**Unadjusted**	**IPW-adjusted**
Age	24.17	7.27
Sex	-4.33	-7.20
Body mass index, kg/m^2	-27.18	1.67
Hypertension	9.28	6.92
Diabetes	19.72	2.67
Dyslipidemia	-13.03	2.96
History of myocardial infarction	26.64	4.13
History of percutaneous coronary intervention	29.21	4.93
History of cerebrovascular accident	4.25	-0.13
STEMI	51.17	5.41
Hemoglobin, g/dL	-21.37	-4.13
Platelet, 10^3/uL	12.37	0.84
Anterior infarction	19.91	8.50
Multi-vessel disease	17.14	1.53
Pre-PCI TIMI flow ≤1	24.02	-1.26
Post-PCI TIMI flow 3	10.58	-1.07
Thrombus aspiration	15.44	1.19
Number of implanted stent	-2.46	-0.63
Stent diameter <3 mm	23.55	-0.94
Aspirin	0	0
P2Y12 Inhibitor	2.92	2.46
Beta-blocker	-4.31	5.32
ACE inhibitor or ARB	-16.61	-1.95

Statin	-13.54	-0.44

Abbreviations: *ACE* angiotensin converting enzyme, *ARB* angiotensin receptor blocker, *IPW* inverse probability weighting, *NLR* neutrophil to lymphocyte ratio, *PCI* percutaneous coronary intervention, *STEMI* ST-segment elevation myocardial infarction, *TIMI* thrombolysis in myocardial infarction.

(2) Supplementary Figures and Figure legends

Supplementary Figure 1. Late gadolinium enhancement and T2-weighted images of an acute myocardial infarction patient.

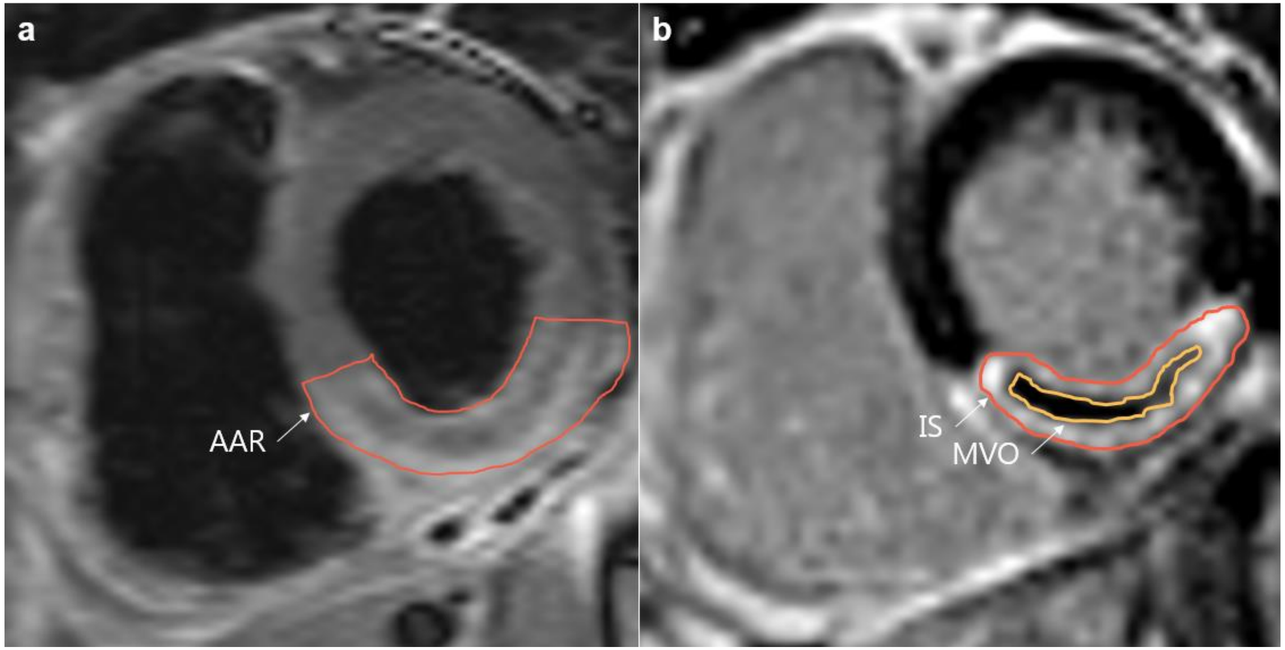

Representative cardiac magnetic resonance images of an acute myocardial infarction patient after revascularization. Areas shown in each figure are (A) area at risk and (B) infarct size and microvascular obstruction.

Abbreviations: *AAR* area at risk, *IS* infarct size, *MVO* microvascular obstruction

Supplementary Figure 2. ROC curves between pre- and post-leukocyte count and large size infarction (≥20%)

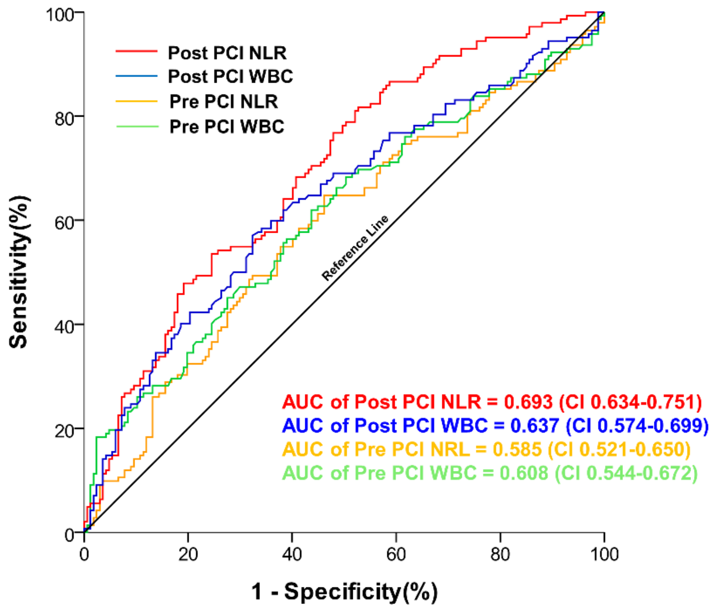

ROC curve analyses were presented to compare the discriminative accuracy of WBC and NLR before and after PCI to predict large size infarction.

Abbreviations: *NLR* neutrophil to lymphocyte ratio, *PCI* percutaneous coronary intervention, *ROC* receiver-operator-characteristic, *WBC* white blood cell counts

Supplementary Figure 3. Cumulative incidence of clinical outcomes at 2-year

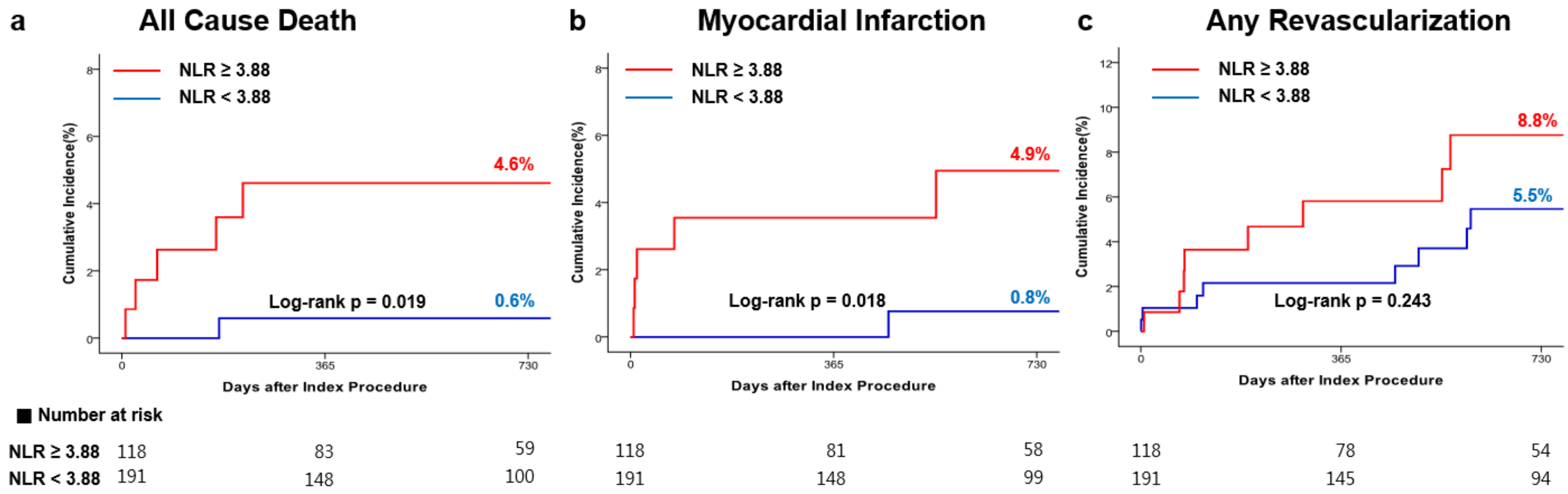

Kaplan-Meier curves are presented to compare the cumulative incidence of (A) all cause death, (B) myocardial infarction, and (C) any revascularization according to NLR 3.88.

Abbreviations: *NLR* neutrophil to lymphocyte ratio

Supplementary Figure 4. Cardiac MRI findings in STEMI patients

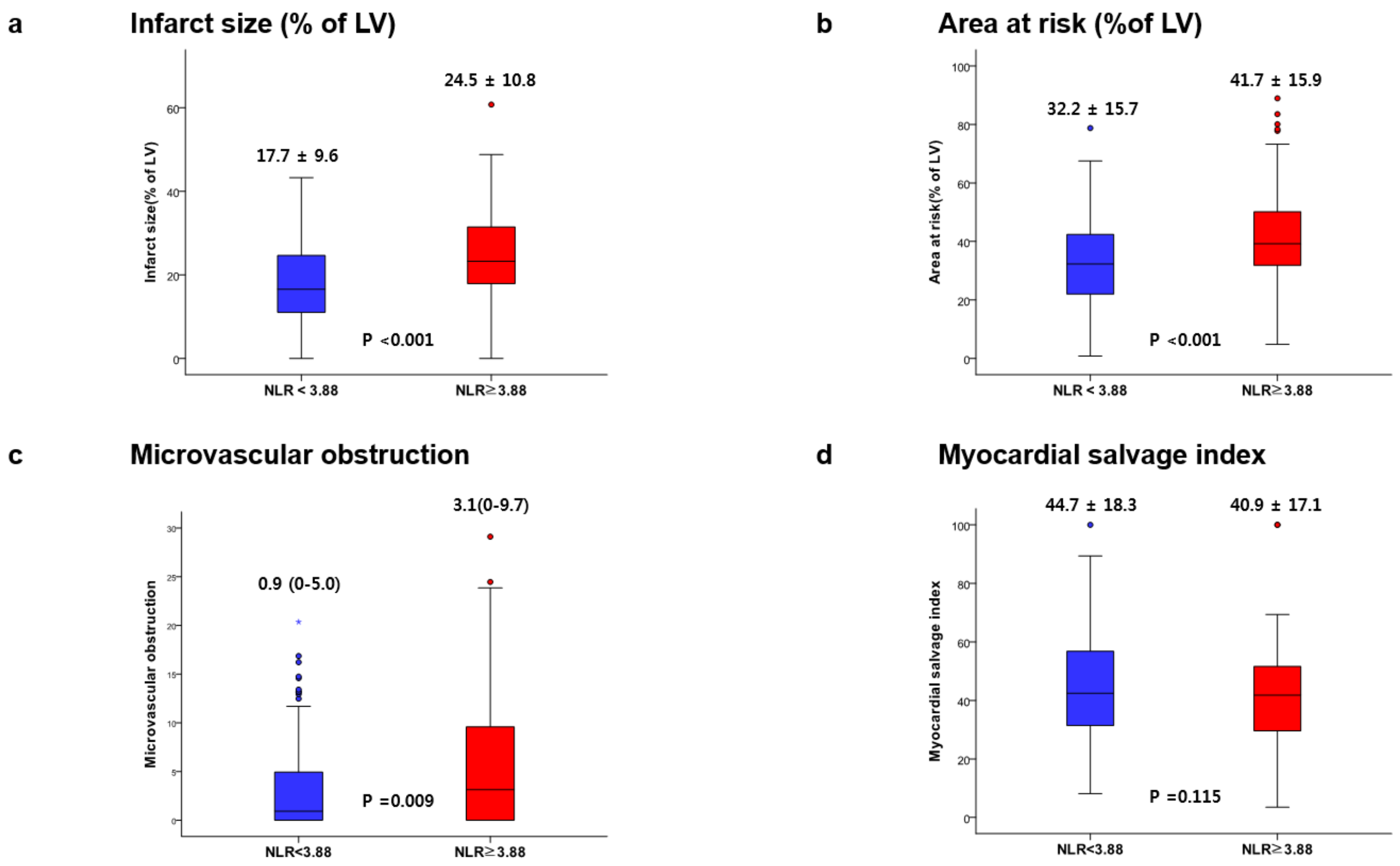

Box-whisker plots are presented to compare the extent of (A) infarct size, (B) area at risk, (C) microvascular obstruction, and (D) myocardial salvage index in STEMI patients according to NLR 3.88.

Abbreviations: *LV* left ventricle, *MRI* magnetic resonance imaging, *NLR* neutrophil to lymphocyte ratio, *STEMI* ST-segment elevation myocardial infarction

Supplementary Figure 5. Cumulative incidence of major adverse cardiac events (MACE) in STEMI patients at 2-year

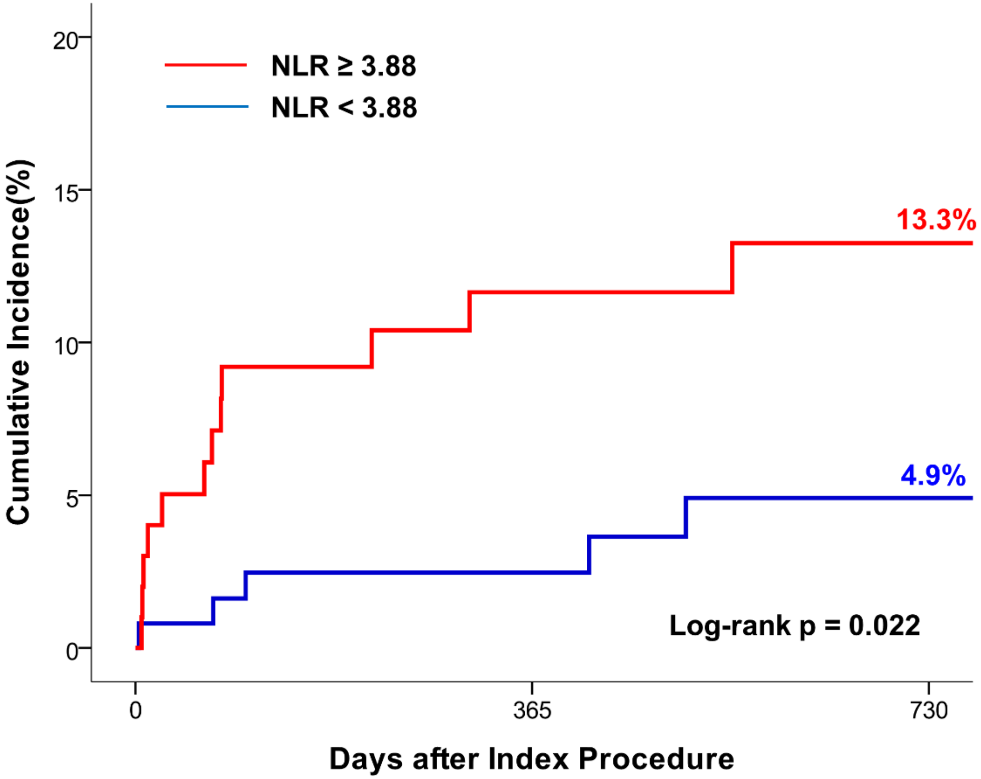

■ **Number at risk**

**NLR ≥ 3.88**	102	67	47
**NLR < 3.88**	125	93	59

Kaplan-Meier curves are presented to compare the cumulative incidence of MACE in STEMI patients according to NLR 3.88

Abbreviations: *MACE* major adverse cardiac event, *NLR* neutrophil to lymphocyte ratio, *STEMI* ST-segment elevation myocardial infarction
